# Maternal and Perinatal Outcomes in Placenta Previa: A Comprehensive Review of Evidence

**DOI:** 10.7759/cureus.59737

**Published:** 2024-05-06

**Authors:** Shreya A Sahu, Deepti Shrivastava

**Affiliations:** 1 Obstetrics and Gynaecology, Jawaharlal Nehru Medical College, Datta Meghe Institute of Higher Education and Research, Wardha, IND

**Keywords:** placenta previa, complications, management strategies, diagnosis, perinatal outcomes, maternal outcomes

## Abstract

Placenta previa poses significant risks to maternal and perinatal health, yet its management remains challenging. This comprehensive review synthesizes current evidence on maternal and perinatal outcomes in placenta previa, addressing its epidemiology, pathophysiology, diagnosis, and management strategies. Placenta previa complicates pregnancies, with increasing incidence linked to factors such as advanced maternal age and rising cesarean rates. Maternal complications, including hemorrhage and placenta accreta spectrum disorders, pose substantial risks. At the same time, perinatal outcomes are marked by increased rates of preterm birth, intrauterine growth restriction, and neonatal morbidity and mortality. Timely diagnosis and appropriate management, including antenatal corticosteroids and multidisciplinary care, are critical for optimizing outcomes. Future research should focus on improving diagnostic methods, evaluating novel interventions, and assessing long-term neurodevelopmental outcomes. This review underscores the importance of informed clinical practice and ongoing research efforts to enhance outcomes for women and infants affected by placenta previa.

## Introduction and background

Placenta previa is a condition characterized by the abnormal implantation of the placenta over or near the internal cervical os (cervical opening), resulting in potential complications during pregnancy and childbirth. It is often categorized based on the degree of coverage of the cervical os, ranging from marginal to complete [1]. The incidence of placenta previa varies globally, but it is generally reported to occur in approximately 0.5-1% of pregnancies. However, its prevalence has been increasing in recent years, possibly influenced by factors such as advanced maternal age and rising rates of cesarean deliveries [2].

Maternal and perinatal outcomes in placenta previa are of paramount importance due to the potential for significant morbidity and mortality for both the mother and the fetus. Maternal complications may include severe hemorrhage, placenta accreta spectrum (PAS) disorders, and the need for emergency cesarean section, which can lead to adverse outcomes such as hysterectomy or even maternal death [3]. Similarly, perinatal complications such as preterm birth, intrauterine growth restriction (IUGR), and neonatal morbidity and mortality are common in pregnancies complicated by placenta previa, highlighting the critical need for effective management strategies [4].

This comprehensive review aims to synthesize existing evidence on maternal and perinatal outcomes in placenta previa, focusing on identifying key risk factors, understanding the underlying pathophysiology, and evaluating current diagnostic and management approaches. By critically analyzing the literature, this review aims to provide clinicians with a comprehensive understanding of the challenges associated with placenta previa and highlights evidence-based interventions to optimize maternal and perinatal outcomes. Ultimately, the review seeks to inform clinical practice and guide future research efforts to improve outcomes for women and infants affected by placenta previa.

## Review

Maternal complications associated with placenta previa

Hemorrhage

Hemorrhage in placenta previa represents a critical concern owing to the potential for substantial blood loss, which can occur from the antenatal period through to the post-cesarean section. Placenta previa stands as a primary cause of third-trimester hemorrhage and manifests through painless bleeding, posing life-threatening risks for both the mother and the infant [5,6]. Patients afflicted with placenta previa face an elevated susceptibility to intrapartum and postpartum massive blood loss, alongside an increased likelihood of placenta accreta, a severe complication associated with maternal mortality and morbidity [7]. Several risk factors contribute to the propensity for hemorrhage in placenta previa, including previous cesarean section, multiparity, advanced maternal age, history of prior placenta previa, prior uterine surgery, and smoking [7]. The prevalence of placenta previa has surged due to escalating rates of cesarean sections and advancing maternal age, underscoring the importance of identifying risk factors and implementing appropriate management strategies to address blood loss during delivery [7].

PAS

PAS is a broad term encompassing abnormal trophoblast invasion into the myometrium, extending at times to or through the uterine serosa [8]. This spectrum includes placenta accreta, increta, and percreta, each signifying different depths of invasion into the uterine wall [9,10]. In a typical pregnancy, the placenta adheres to the inner uterine wall without penetrating it, being expected to be expelled shortly after the baby's delivery [9]. Conversely, in individuals affected by PAS, the placenta grows within the uterine wall and may even extend beyond it, invading neighboring organs [10]. This abnormal growth significantly heightens the risk of life-threatening hemorrhage during attempts to remove the placenta manually [10].

The incidence of PAS is escalating, with common risk factors including prior cesarean delivery, previous uterine surgery, and placenta previa [9]. Diagnosis typically arises from suspicions raised during routine prenatal ultrasound, followed by confirmation through a meticulous ultrasound examination conducted by a maternal-fetal medicine specialist [11]. Pelvic MRI is often sought to elucidate further the extent of the placental anomaly and aid in surgical planning [11].

The management of PAS demands a multidisciplinary approach involving a team of experts well-versed in this condition [11]. Delivery for PAS patients is typically scheduled around 34 to 35 weeks of gestation, with hysterectomy being the standard course of action in most instances, albeit with the preservation of ovaries being almost invariably pursued [11]. The team at the NewYork-Presbyterian (NYP)/Columbia University Irving Medical Center (CUIMC) stands as one of the nation's most experienced in PAS management. It actively engages in research to enhance outcomes for individuals grappling with this condition [11].

Maternal Morbidity and Mortality

Placenta previa stands as a significant contributor to bleeding episodes during the latter half of pregnancy, delivery, and the postpartum period, presenting a complication with profound implications for maternal-perinatal morbidity and mortality [12]. While the global incidence of placenta previa is estimated at 0.25-0.50%, it can soar to 5.1% in regions such as the Americas and the Caribbean [13]. Hemorrhage remains the foremost cause of maternal mortality worldwide (27%), emphasizing the public health significance of placenta previa [13]. In 2014, Colombia reported 493 maternal deaths, with 27 attributed to first-trimester bleeding, eight to second-trimester bleeding (one linked to placenta previa complications), and 46 to postpartum hemorrhage [13].

Placenta previa engenders maternal morbidity, encompassing severe hemorrhage, hypovolemic shock, fetal hypoxia, preterm labor or delivery, and neonatal health risks associated with premature birth, emergent cesarean delivery, and the potential need for a hysterectomy if placental detachment proves problematic [14]. Additionally, it poses a risk of blood loss for the infant and potential fatality [14]. Risk factors for placenta previa include previous cesarean section, prior uterine surgeries (e.g., myomectomy, uterine curettage, or manual placental removal), advanced maternal age, multiparity, multiple pregnancies, uterine myxomatosis, smoking, assisted reproductive treatments, and ethnicity (Black or Asian) [14,15].

The management of placenta previa necessitates a multifaceted approach involving various specialties. Early identification of risk factors and strategic management strategies promise to improve maternal and fetal outcomes [14]. Conservative management is reserved for cases involving fertility aspirations and extensive disease due to surgical complexity [14].

Impact on Obstetric Management

The impact of placenta previa on obstetric management is substantial, necessitating careful consideration to achieve optimal maternal and perinatal outcomes. Placenta previa poses a heightened risk of maternal hemorrhage, often requiring emergency peripartum hysterectomy as a lifesaving intervention [16]. The obstetric management decisions are influenced by the particular type of placenta previa, whether it's complete, incomplete, marginal, or low-lying [16]. Moreover, the number of prior cesarean deliveries contributes to assessing obstetric hysterectomy risk in women with placenta previa [16].

The diagnosis of placenta previa holds significant implications for obstetric management, shaping pregnancy care and outcomes [17]. Management objectives include monitoring placental position, ruling out PAS disorders, minimizing bleeding risk, and determining the appropriate level of monitoring, whether inpatient or outpatient [18]. Delivery timing assumes critical importance in placenta previa cases, with recommendations favoring late preterm delivery between 36 weeks zero days and 37 weeks zero days of gestation to balance bleeding risks against the dangers of prematurity [18]. For cases involving invasive placentations such as accreta, increta, or percreta, meticulous planning and preparation before delivery are imperative due to their elevated mortality and morbidity rates [18].

**Figure 1:**
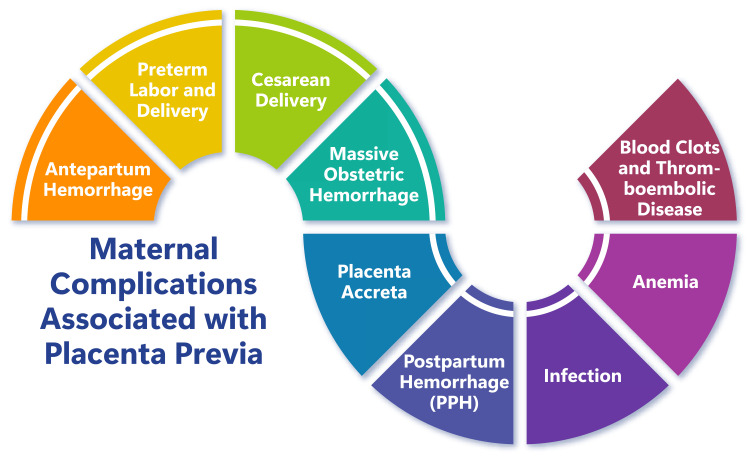
Maternal complications associated with placenta previa Figure credit: Dr. Shreya Sahu

Effective blood loss control forms a cornerstone of obstetric management in complex pregnancies, with interventions such as balloon catheters for angiographic embolization of pelvic vessels and aortic balloon occlusion preceding cesarean hysterectomy (C-HYST) deployed to mitigate hemorrhage [19]. C-HYST may become unavoidable in instances of complete placenta previa coupled with a history of cesarean deliveries, underscoring the necessity for tailored management plans based on individual patient circumstances [19]. Figure 1 shows maternal complications associated with placenta previa.

Perinatal complications associated with placenta previa

Preterm Birth

Preterm birth emerges as a prevalent complication linked to placenta previa, with approximately 5% of all preterm deliveries attributed to this condition [20]. Patients with placenta previa face a notably elevated risk of preterm birth compared to those without this condition [20,21]. Antepartum bleeding serves as a robust predictor of preterm delivery in pregnancies complicated by placenta previa [22]. Furthermore, individuals with placenta previa who deliver prematurely exhibit an augmented susceptibility to recurrent spontaneous preterm birth, irrespective of placental location [23]. This risk escalates further among those delivering preterm before 34 weeks of gestation [23]. Notably, women with placenta previa delivering prematurely, mainly before 34 weeks of gestation, face an increased risk of recurrent spontaneous preterm birth, regardless of placental implantation site [23]. Consequently, strict monitoring by high-risk pregnancy specialists is advisable for these patients [23].

IUGR

Preterm birth is a prevalent complication associated with placenta previa, accounting for approximately 5% of all preterm deliveries [20]. Patients with placenta previa exhibit a significantly heightened risk of preterm birth compared to those without this condition [20,21]. Antepartum bleeding emerges as a robust predictor of preterm delivery in pregnancies complicated by placenta previa [22]. Moreover, individuals with placenta previa who deliver prematurely are at increased risk of recurrent spontaneous preterm birth, regardless of the location of the placenta [23]. This risk is especially notable among individuals delivering before 34 weeks of gestation [24]. Notably, women with placenta previa delivering prematurely, especially before 34 weeks of gestation, face an elevated risk of recurrent spontaneous preterm birth, irrespective of the placental implantation site [25]. Thus, stringent monitoring by high-risk pregnancy specialists is recommended for these patients [25].

Neonatal Morbidity and Mortality

Neonatal morbidity and mortality, associated with placenta previa, pose significant concerns for healthcare providers. Studies indicate that the neonatal mortality rate is markedly higher in pregnancies complicated by placenta previa compared to those without this condition. For instance, a study revealed a neonatal mortality rate of 10.7 per 1,000 births in previa cases, contrasting with 2.5 per 1,000 in other pregnancies (relative risk: 4.3; 95% confidence interval: 4.0, 4.8) [26,27]. Additionally, perinatal mortality rates are elevated in placenta previa, with one study reporting a rate of 81 per 1,000 births [3]. The heightened risk of neonatal morbidity and mortality stems from various factors, including preterm birth, low birth weight, birth asphyxia, and neonatal sepsis [28]. Prematurity notably contributes to perinatal mortality, with one study attributing 42.85% of perinatal mortality cases to prematurity [28]. Furthermore, respiratory distress syndrome (RDS) and asphyxia also play significant roles in perinatal mortality associated with placenta previa [28]. The literature underscores the importance of a multidisciplinary approach in managing placenta previa and PAS disorders, with early identification of risk factors and strategic management being pivotal for improving maternal and fetal outcomes [28]. Conservative management should be considered for cases involving fertility desires and extensive disease due to surgical complexities [28].

Long-Term Neurodevelopmental Outcomes

**Figure 2:**
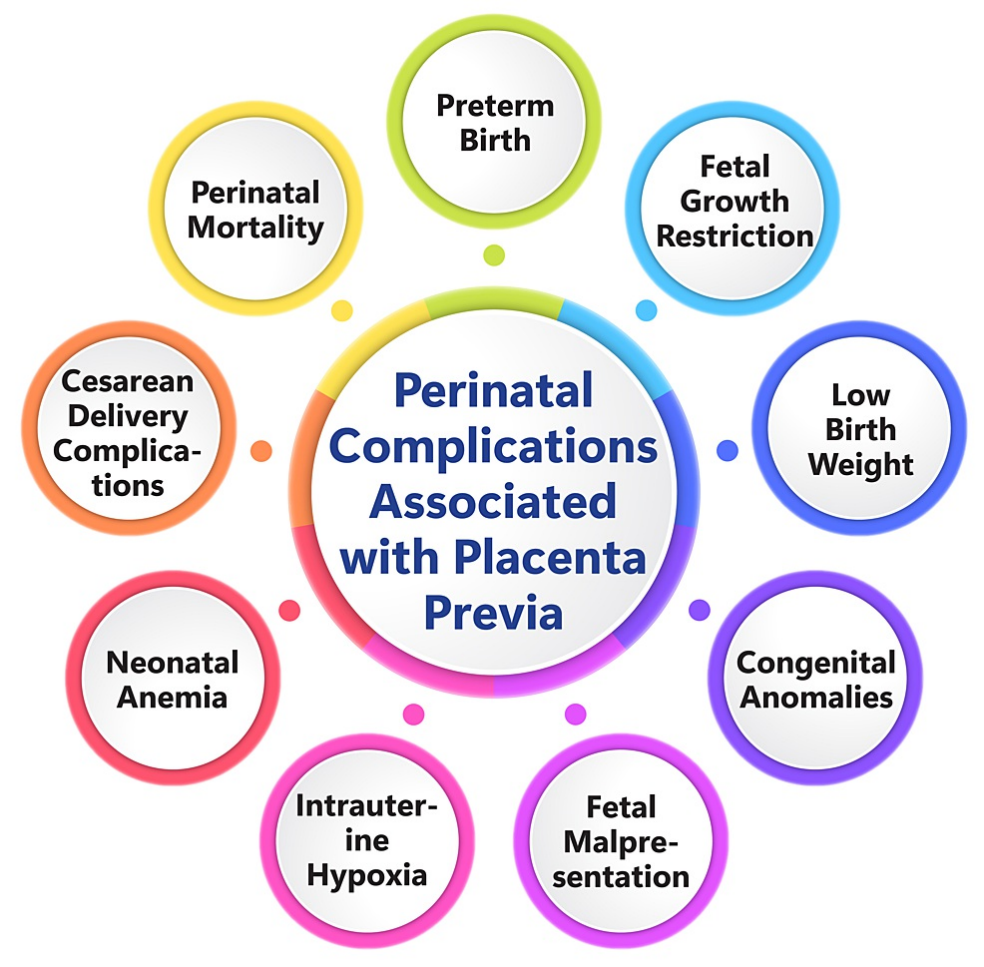
Perinatal complications associated with placenta previa Figure credit: Dr. Shreya Sahu

The long-term neurodevelopmental outcomes of infants born with placenta previa represent an essential area of study. Placenta previa's association with IUGR underscores its potential impact on neurodevelopment, as IUGR has been linked to poorer outcomes, compared to infants with appropriate fetal growth [29]. Furthermore, IUGR serves as a risk factor for emotional and behavioral disorders, with the underlying cause of growth restriction playing a significant role in assessing the risk of adverse outcomes [29]. Placenta accreta, often concurrent with placenta previa, introduces delivery complications and may necessitate preterm cesarean delivery, which has been associated with an increased risk of neurodevelopmental disorders [30]. However, a population-based study revealed that pregnancies with placenta previa reaching term do not substantially elevate the risk of future health issues in children [30]. Nevertheless, it is crucial to acknowledge that research on the long-term neurodevelopmental outcomes of infants born with placenta previa remains ongoing. Figure 2 shows perinatal complications associated with placenta previa.

Diagnosis and management of placenta previa

Antenatal Diagnosis

Antenatal diagnosis is crucial in routine clinical practice for identifying life-limiting conditions and genetic disorders, offering early intervention and management opportunities. Utilizing ultrasound, cytogenetic tests, and MRI, antenatal diagnosis can detect approximately 90% of lethal or life-limiting conditions during the first and second trimesters [31]. Specifically, ultrasound is a valuable tool for antenatal diagnosis of placenta accreta, enabling the identification of invasive or adherent placenta with the absence of decidua between the myometrium and placenta [32]. This early diagnosis of placenta accreta can mitigate peripartum blood loss and reduce the necessity for blood transfusions [33].

Additionally, antenatal diagnosis is pivotal in identifying biliary atresia (BA), a congenital liver disease affecting the bile ducts. While the etiology and pathogenesis of BA remain primarily unknown, certain features can be detected during routine antenatal ultrasound examinations at various gestational ages, including non-visualization of the fetal gallbladder (GB), cystic dilatation of the extrahepatic biliary tract, and associated congenital anomalies [34]. Although the impact of antenatal diagnosis on surgical outcomes for BA remains unexplored mainly, early and accurate postnatal assessments can prevent diagnostic and surgical delays, facilitating early diagnosis and prompt portoenterostomy, thereby improving outcomes, particularly for cystic and biliary atresia splenic malformation (BASM) subgroups [34].

Imaging Modalities

The diagnosis of placenta previa primarily relies on ultrasound, renowned as the gold standard for detecting this condition [35]. Ultrasound is a safe, cost-effective, and accurate imaging modality, proficient in assessing placental location and the distance between the placental edge and the internal cervical os [36]. Transvaginal ultrasonography proves particularly valuable for diagnosing placenta previa, enabling meticulous examination of the lower uterine segment and cervix [36].

While ultrasound serves as the primary diagnostic tool, magnetic resonance imaging (MRI) may also be employed, especially in cases where ultrasound findings are inconclusive, or when there's a high suspicion of placenta accreta, a more invasive form of placental implantation [37]. MRI provides additional insights into the degree of placental invasion and the relationship between the placenta and surrounding structures like the bladder [37]. However, due to its higher cost and limited accessibility compared to ultrasound, MRI isn't routinely utilized for diagnosing placenta previa [37].

Furthermore, ultrasound is crucial in diagnosing placenta accreta, characterized by abnormal placental adherence to the uterine wall [37]. It aids in identifying signs of placental invasion, such as loss of the normal hypoechoic zone between the placenta and the myometrium, increased vascularity at the placental-myometrial interface, and focal interruptions in the myometrium [37]. MRI may also be utilized for diagnosing placenta accreta, particularly in cases where ultrasound findings are inconclusive, or when there's a high suspicion of more invasive forms of placental implantation like placenta increta or placenta percreta [37].

Conservative Management vs. Surgical Interventions

The management of placenta previa encompasses two main approaches: conservative management and surgical interventions. Conservative management involves prophylactic double bilateral ligation of uterine arteries before placental removal, followed by tamponade using a saline-filled balloon catheter [38]. This strategy aims to circumvent hysterectomy and safeguard the patient's future fertility [38]. Conversely, surgical interventions typically entail C-HYST, historically the standard treatment for placenta previa [39]. However, there has been a gradual shift toward conservative management over the past decade, driven by the desire to mitigate maternal severe morbidity and preserve fertility [39]. The choice between conservative management and surgical interventions hinges on factors such as the extent of placental invasion, the patient's fertility preferences, and the availability of resources and facilities for managing placenta previa [39].

A double setup examination may be conducted when the placental location remains uncertain. Here, one management team prepares for an uncomplicated vaginal delivery while a second team stands ready for immediate surgery [40]. Invasive placentations, such as accreta, increta, or percreta, pose significant mortality and morbidity risks, necessitating delivery plans that include patient-specific blood matching and informed consent for the potential predelivery placement of balloon catheters for angiographic embolization of pelvic vessels, as well as aortic balloon occlusion before C-HYST and other hemorrhage control measures [40]. Consultations with specialists in obstetrics, obstetric anesthesiology, interventional radiology, surgical oncology or general surgery, gynecologic oncology, urology, and other pertinent fields may be indispensable for effective placenta previa management [40].

Timing and Mode of Delivery

The timing and method of delivery for patients with placenta previa are pivotal in mitigating the risk of maternal and fetal complications. According to the Society of Obstetricians and Gynaecologists of Canada (SOGC) guideline, for uncomplicated complete placenta previa, scheduling delivery between 36 and 37 weeks is advisable [17]. This recommendation stems from available data indicating that this timing minimizes labor-related bleeding risk while reducing fetal prematurity risks [17]. However, earlier delivery might be warranted based on individual bleeding profiles, prior bleeding history, or signs of preterm labor [40]. After 35 weeks gestation, the distance between the placental edge and internal cervical os on transvaginal ultrasonography becomes a crucial factor in determining the delivery mode, with placental edge-to-cervical os distances of less than 2 cm typically associated with a higher cesarean rate [40].

During cesarean delivery for placenta previa, a low transverse uterine incision is commonly preferred. However, a vertical uterine incision may be considered in cases of an anterior placenta due to the risk of fetal bleeding [40]. Invasive placental implantations, such as placenta accreta, increta, or percreta, represent severe maternal complications linked with placenta previa. If a patient is deemed at increased risk for invasive placentation, the patient and the surgical team must be adequately prepared before delivery. These invasive placental conditions carry substantial mortality (7% with placenta accreta) and morbidity risks (including blood infection and adjacent organ damage) [40].

Controlling blood loss is paramount in managing these complex pregnancies, with delivery plans typically incorporating patient-matched blood and obtaining informed consent for possible predelivery placement of balloon catheters for angiographic embolization of pelvic vessels [40]. Other hemorrhage control measures may include B-Lynch or parallel vertical compression sutures, uterine artery ligation (O'Leary stitch), and aortic balloon occlusion before C-HYST [40].

Evidence-based interventions and strategies for improving outcomes

Antenatal Corticosteroids (ACS)

ACS are medications administered to pregnant women anticipating preterm birth, proving highly effective in improving outcomes for preterm newborns by reducing the incidence and mortality of infant RDS, a potentially life-threatening condition stemming from underdeveloped lungs [41]. The benefits of ACS extend beyond RDS, encompassing a reduction in intraventricular hemorrhage, necrotizing enterocolitis, and systemic infections within the first two days of life [42]. Recognizing these benefits, the World Health Organization (WHO) recommends a single course of ACS for women at risk of preterm birth (less than 34 weeks of gestation) to mitigate the risk of child mortality, irrespective of resource level [43,44].

The efficacy of ACS was initially established in a landmark randomized controlled trial employing betamethasone in 1972 by Sir Graham Liggins and Ross [45]. ACS has since demonstrated a capacity to decrease the risk of late miscarriages, infant deaths, RDS, intraventricular hemorrhage, necrotizing enterocolitis, and systemic infections within the first two days of life [45]. Notably, ACS administration does not seem to elevate the incidence of fetal membrane infection or chorioamnionitis [46]. Moreover, evidence suggests that administering ACS to patients with preterm premature rupture of membranes (PPROM) yields similar reductions in neonatal complications, akin to its effects on preterm birth [46]. The optimal timing and dosing of ACS administration remain subjects of ongoing research. However, prevailing guidelines propose that women nearing viability at delivery should be considered for ACS administration [47].

Antenatal Surveillance

Antenatal surveillance plays a pivotal role in managing placenta previa, with evidence-informed practices guiding the optimization of surveillance protocols to enhance perinatal outcomes and minimize harm [17]. While close surveillance of fetal growth is recommended, the data regarding the association between placenta previa and fetal growth abnormalities are inconclusive [48]. Nevertheless, maintaining vigilance, adhering to a systematic approach, and following standardized protocols are imperative to ensure optimal outcomes in placenta previa management [48].

The phenomenon of placental "migration" away from the internal os as the lower uterine segment develops, underscores the dynamic nature of placental positioning. Research suggests a migration rate of approximately 5.4 mm per week, with over 98.4% of suspected low-lying/placenta previa cases in the second trimester resolving before delivery, typically around 26 weeks of gestation, leaving only 1.6% persisting until term [48]. Ultrasound emerges as a critical tool for placental localization, with various techniques available to assess the placenta, lower uterine segment, and cord insertion to screen for placenta previa [48].

Accurate identification of abnormal placentation is crucial for determining appropriate management strategies in at-risk pregnancies and minimizing morbidity and mortality risks for both mother and child. The management of placenta previa typically involves several interventions, including avoidance of digital vaginal examination, delaying delivery until 36 weeks gestation and documented fetal lung maturity, transfusion support to maintain maternal hematocrit at or above 30%, serial ultrasonography, antepartum fetal heart rate monitoring, administration of glucocorticoids, tocolytic therapy, and elective cesarean section delivery [49]. Placenta previa diagnosis primarily relies on ultrasound, either during routine prenatal appointments or following an episode of vaginal bleeding [49]. Any patient with suspected or confirmed placenta previa and new-onset vaginal bleeding should be promptly admitted to the hospital for close monitoring [40]. The focus of care should prioritize maternal hemodynamic stability and fetal well-being, with ultrasound and sterile speculum examination utilized to assess the quantity and source of bleeding [40].

Role of Multidisciplinary Team

A multidisciplinary team's involvement in managing placenta previa and PAS disorders is pivotal in enhancing maternal and perinatal outcomes. Evidence underscores the efficacy of multidisciplinary teams in reducing severe maternal morbidity among women with invasive placenta previa and improving outcomes in PAS patients [39,50]. This collaborative approach entails the integration of healthcare professionals from diverse specialties, including maternal-fetal medicine, anesthesiology, neonatology, urology, and experienced surgeons in PAS management [50]. By leveraging the expertise of each team member, tailored management plans can be developed for individual patients, optimizing surveillance protocols, and ensuring readiness for both planned and unforeseen cases of PAS [50]. Research indicates that multidisciplinary teams contribute significantly to improved maternal outcomes in PAS cases, often resulting in a higher likelihood of uterine preservation and a reduced risk of hysterectomy [50]. Moreover, implementing checklists and standardized procedures for C-HYST has been associated with favorable maternal outcomes, including reduced blood loss, decreased blood transfusion requirements, and lower intensive care unit admissions [51]. This systematic approach enhances patient safety and streamlines clinical processes, promoting efficiency and consistency in care delivery.

## Conclusions

In conclusion, this comprehensive review has underscored the significant maternal and perinatal implications associated with placenta previa. It has elucidated the heightened risks of maternal complications, including hemorrhage and PAS disorders, alongside the increased likelihood of adverse perinatal outcomes such as preterm birth and neonatal morbidity. These findings emphasize the critical importance of timely diagnosis and effective management strategies in mitigating the risks posed by placenta previa. Clinicians must remain vigilant, employ close antenatal surveillance, consider interventions like ACS, and engage multidisciplinary teams to ensure comprehensive care. Furthermore, this review highlights the imperative of patient education and support to facilitate informed decision-making. Looking ahead, further research is warranted to explore novel diagnostic methods, assess the efficacy of emerging interventions, and investigate long-term outcomes for both mothers and infants affected by placenta previa. By advancing our understanding and refining clinical practices, we can strive to optimize outcomes and enhance the well-being of women and infants impacted by this complex obstetric condition.
